# Prospective Study of Intense Pulsed Light With Postoperative Radiotherapy for Keloids in Young and Middle‐Aged Men

**DOI:** 10.1111/jocd.70567

**Published:** 2025-11-27

**Authors:** Qiaoling Weng, Junjun Sang, Jie Wu, Wen Xu, Shengping Chen, Xiangqi Chen

**Affiliations:** ^1^ 900th Hospital of PLA Joint Logistic Support Force Fuzhou China

**Keywords:** intense pulsed light, keloids, radiotherapy, surgical excision

## Abstract

**Objective:**

To evaluate the efficacy of using a combination of intense pulsed light (IPL) with a 500–600 nm wavelength filter and postoperative radiotherapy to treat chest scars in young and middle‐aged men.

**Methods:**

A prospective randomized controlled trial was conducted with 52 male patients. Those receiving postoperative radiotherapy combined with IPL were assigned to the observation group, while those receiving only postoperative radiotherapy were assigned to the control group. Follow‐ups were conducted from 0 to 24 months to assess efficacy using the Vancouver Scar Scale (VSS) and the Patient and Observer Scar Assessment Scale (POSAS).

**Results:**

No significant differences in VSS or POSAS scores were observed prior to treatment (*p* > 0.05). Following treatment, however, the observation group demonstrated significantly superior outcomes (*p* < 0.001), exhibiting lower scores in terms of color, vascular distribution, softness and total VSS (*p* < 0.05), though thickness scores remained unchanged (*p* > 0.05). Pain and itchiness scores were also significantly lower in the observation group.

**Conclusion:**

Combining postoperative radiotherapy with IPL is more effective than radiotherapy alone at improving chest scars, and significantly alleviates pain and itching symptoms in young and middle‐aged men.

## 1 Background

Scarring refers to alterations in the appearance and histopathological structure of normal skin tissue resulting from various types of injury to the skin. It is a natural by‐product of the body's wound‐healing process [1]. However, an imbalance may occur during wound healing between collagen synthesis and degradation, leading to pathological scarring. This includes hypertrophic scars and keloids, which are abnormal outcomes of wound healing [2, 3]. These conditions may be caused by trauma, folliculitis, and the high prevalence of inflammatory diseases such as acne [4]. Not only do keloids affect the patient's appearance, they are also often accompanied by varying degrees of itching and pain. They are difficult to treat, have a high recurrence rate and create significant psychological distress for patients, severely impacting their quality of life [5].

Keloids on the chest are notoriously difficult to treat and are more likely to recur than keloids in other parts of the body [6]. The most widely accepted and effective treatment method currently involves surgical excision combined with radiotherapy [7]. However, the cure rate for chest keloids remains low. In recent years, several researchers have suggested that phototherapy could be beneficial for keloids [8]. Specifically, this study selected intense pulsed light (IPL) therapy with a wavelength filter of 500–600 nm. Unlike conventional IPL (broad spectrum, 400–1200 nm) or lasers (monochromatic light), the focused wavelength range of IPL preferentially targets hemoglobin in keloid microvessels while minimizing melanin absorption. This enhances safety in pigmented skin and may more effectively suppress the pro‐fibrotic signaling pathways implicated in keloid recurrence [9, 10]. Despite these potential advantages, rigorous comparative studies evaluating phototherapy (particularly IPL) combined with surgical excision and radiotherapy are lacking. This study aims to compare the efficacy of IPL combined with postoperative radiotherapy with that of postoperative radiotherapy alone. The follow‐up period has been extended to two years in order to determine whether IPL's mechanistic advantages translate into reduced recurrence and improved clinical outcomes.

## 2 Materials and Methods

### 2.1 Patients and Study Design

We conducted a prospective randomized controlled trial, selecting a total of 52 male patients with chest keloids who visited the 900th Hospital of Joint Logistic Support Force hospital between March 2018 and March 2019. The study was approved by the Joint Logistics Support Force 900th Hospital Ethics Committee (2017‐030) and was conducted in accordance with the principles of the revised Declaration of Helsinki. Written informed consent was obtained from all participants prior to the study.

**FIGURE 1:**
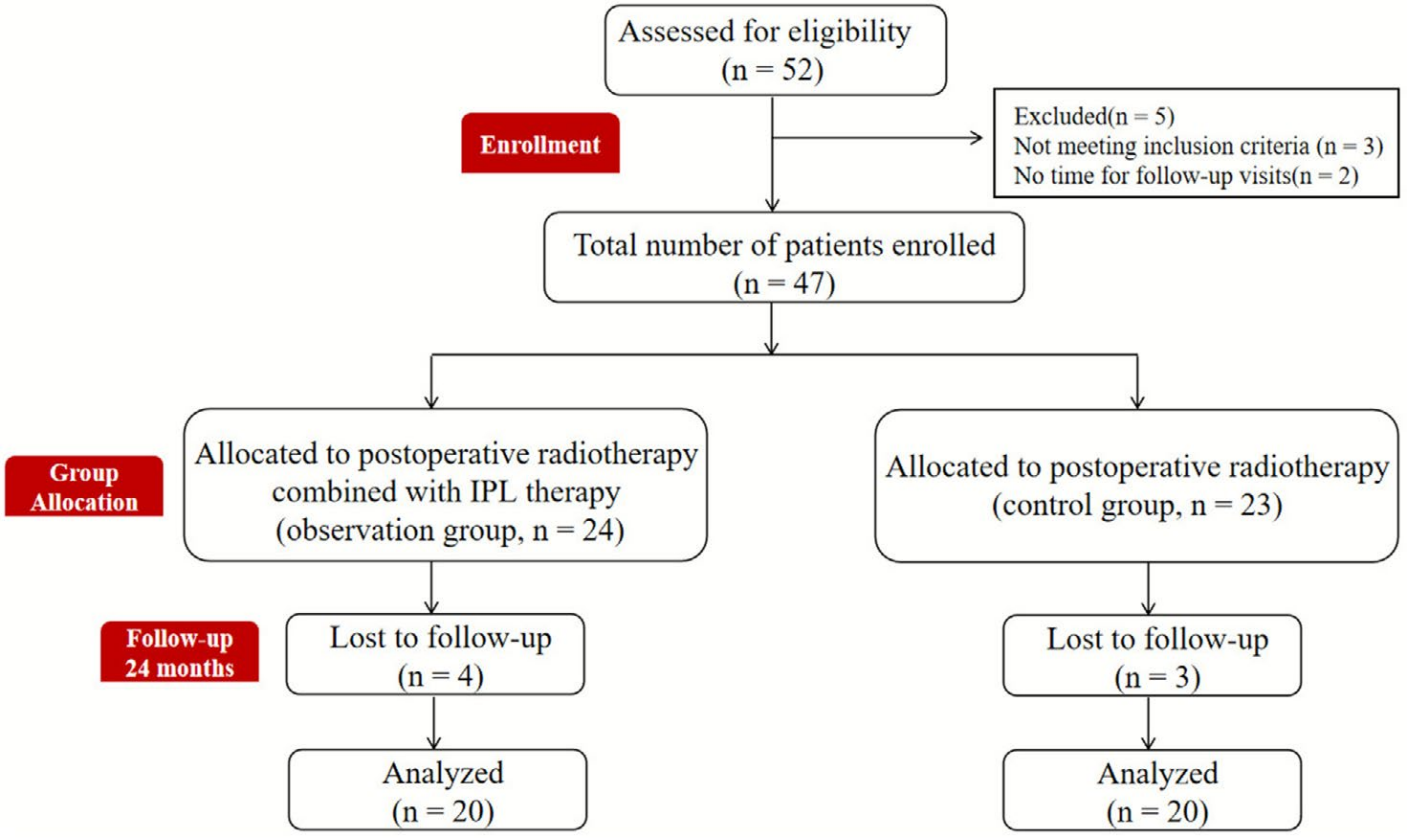
Flow diagram of participants.

Five patients were excluded based on the inclusion and exclusion criteria, as well as their unwillingness to participate in follow‐up assessments. The remaining patients were randomly assigned to either the postoperative radiotherapy combined with IPL group (observation group, *n* = 24) or the postoperative radiotherapy alone group (control group, *n* = 23). Seven patients were lost to follow‐up (four from the observation group and three from the control group), leaving 40 patients who completed the study (20 in each group) (Figure 1).

The inclusion and exclusion criteria are summarized in Table 1.

**TABLE 1:** Inclusion and exclusion criteria.

Category	Criteria
Inclusion	Male patients aged 20–30 years with keloid scars on the chest Diagnosis confirmed via clinical examination or histopathological analysis No invasive treatment received in the past six months Keloid dimensions: length 1–5 cm, width 1–3 cm No coagulation disorders No contraindications for radiotherapy or IPL No history of photosensitivity Willing to adhere to treatment protocols and attend regular follow‐up appointments Provided informed consent with commitment to complete 24 months of follow‐up
Exclusion	Patients with other serious systemic diseases or poor overall health Patients unable to adhere to treatment or lost to follow‐up Patients with a pathological diagnosis other than keloids

Patients who met the inclusion criteria were randomly assigned to groups based on their voluntary agreement. The randomization method was as follows: a random number generation table was created using SAS statistical software based on a 1:1 ratio. These random numbers were then placed in sealed envelopes and drawn in the order of patient enrolment. This process randomly assigned patients to either the IPL combined with postoperative radiotherapy group (observation group) or the postoperative radiotherapy alone group (control group). To minimize potential bias, this study employed an assessor‐blinded methodology. Those responsible for evaluating treatment efficacy (including VSS and POSAS scoring and statistical analysis) were blinded to the group allocation and specific treatment protocols of the patients throughout the study.

### 2.2 Treatment Methods

Surgical procedure: Direct excision was performed for keloids measuring less than 2 cm in length. For keloids larger than 2 cm, a ‘Z’ or ‘W’ flap plasty was used. All surgical procedures used PDS antimicrobial sutures for subcutaneous and skin closure, with the sutures being removed two weeks after the operation.

Radiotherapy protocol: Postoperative radiotherapy commenced within 24 h of surgery using the Varian TrueBeam radiotherapy system. A 9 MeV electron beam was used to irradiate an area extending 2 cm beyond the surgical incision. A total radiation dose of 20 Gy was delivered in 5 Gy increments over four consecutive days. The irradiated area's shape and size were tailored using lead shielding, and the radiotherapy plan was developed by the physics department.

IPL treatment: After the scar area had been cleansed, the patients were provided with protective eyewear and positioned supine. A cold gel, between 5 and 8 mm thick, was then applied to the scar area. Pulsed light treatment was administered using the Alma device with a wavelength filter of 500–600 nm. The pulse width was initially set to 12 ms and the treatment head was held perpendicular to the scar surface to ensure full contact with the cold gel. The endpoint reaction of the skin was monitored until mild erythema was observed in the scar and surrounding tissue. Based on the observed endpoint reaction, adjustments to the pulse width were made, ensuring approximately 10% overlap of the light spots. Once the pulse width had been established, treatment moved on to the next section. The phototherapy equipment used was the Alma Brilliance 360, manufactured by Fotona Medical, operating in the 500–600 nm range. The first IPL session was performed within 48 h after suture removal. Each IPL treatment session was scheduled four weeks apart, totaling four to five sessions.

In the control group, patients underwent surgery combined with radiotherapy. In the observation group, IPL treatment commenced four weeks after the sutures were removed following surgery, with sessions spaced four weeks apart, amounting to four to five sessions.

### 2.3 Primary Endpoint of the Study

The primary objective of this study was to evaluate the clinical efficacy of the treatments administered to both groups, both before and after the intervention. This evaluation was conducted using the Vancouver Scar Scale (VSS) and the Patient and Observer Scar Assessment Scale (POSAS) [9]. The VSS quantifies scar characteristics across four domains: pigmentation (0–3 points), thickness (0–4 points), vascularity (0–3 points) and pliability (0–5 points). The total score ranges from 0 to 15, with higher scores indicating worse outcomes. The POSAS comprises two components: the Patient Scar Assessment Scale (PSAS, six items) and the Observer Scar Assessment Scale (OSAS, six items). Each item is rated on a 10‐point scale (1 = normal skin; 10 = the worst imaginable), generating separate subtotals for the patient's and observer's perspectives (range 6–60 per subscale).

Follow‐up assessments were conducted during treatment and at 0, 1, 2, 3, 4, 5, 6, 8, 10, 12, 15, 18, 21 and 24 months afterwards, primarily via outpatient visits. Efficacy was evaluated by comparing changes in VSS and POSAS scores between the observation and control groups before treatment and throughout the follow‐up period. These scores assessed various aspects of keloids comprehensively, including pigmentation, thickness, vascularity, pliability, pain, itching and overall condition. This provided a thorough reflection of improvement in keloid scars.

Clinical efficacy was assessed based on the following criteria: A scar clearance rate of 50%–74% was considered effective, 75%–99% good, and 100% cured. Failure to meet any of these criteria was categorized as ineffective. The efficacy rate was calculated using the following formula: Effective rate = (number of cured cases + number of good cases + number of effective cases)/total number of cases.

### 2.4 Adverse Reaction Observation

Throughout the treatment process, we recorded any instances of severe pain, erythema, blistering, and incision infection at the treatment site, as well as other adverse reactions such as dizziness and allergic responses.

### 2.5 Statistical Analysis

Data analysis was performed using SPSS version 21.0. Measurement data were presented as mean ± standard deviation, with inter‐group comparisons conducted using the t‐test. Repeated measures data were analyzed with repeated measures ANOVA, while ordered categorical variables were assessed using the Wilcoxon ranked sum test. A two‐tailed test was employed, with a *p*‐value < 0.05 considered statistically significant.

## 3 Results

### 3.1 Baseline Characteristics

This prospective study enrolled 40 young and middle‐aged male patients with chest keloids, evenly allocated to an observation group (*n* = 20) and a control group (*n* = 20). As shown in Table 2, all participants were male, with comparable mean ages (observation: 25.55 ± 2.14 years, control: 25.50 ± 2.04 years; *p* = 0.94) and keloid characteristics: thickness (2.66 ± 0.78 mm vs. 2.61 ± 0.87 mm, *p* = 0.85) and area (4.91 ± 1.87 cm^2^ vs. 5.08 ± 2.23 cm^2^, *p* = 0.80). Surgical techniques included W‐plasty (80% vs. 65%), Z‐plasty (0% vs. 10%), and fusiform incision (20% vs. 25%), with no statistical differences in distribution (*p* > 0.05). Etiologies of keloid formation—accident trauma (55% vs. 45%), folliculitis (45% vs. 50%), and operative incision (0% vs. 5%)—were similarly balanced between groups (*p* > 0.05), confirming homogeneity at baseline.

**TABLE 2:** Demographic characteristics of the study patients.

	Observation group (*n* = 20)	Control group (*n* = 20)	*p*
Male sex, *n* (%)	20 (100%)	20 (100%)	—
Age (years)	25.55 ± 2.14	25.50 ± 2.04	0.94
Keloid thickness (mm)	2.66 ± 0.78	2.61 ± 0.87	0.85
Keloid area (cm^2^)	4.91 ± 1.87	5.08 ± 2.23	0.80
Surgical technique, *n* (%)			
W‐plasty	16 (80%)	13 (65%)	0.41
Z‐plasty	0 (0%)	2 (10%)	
Fusiform incision	4 (20%)	5 (25%)	
Causes of keloid formation, *n* (%)			
Accident trauma	11 (55%)	9 (45%)	0.75
Folliculitis	9 (45%)	10 (50%)	
Operative incision	0 (0%)	1 (5%)	

*Note:* Data are presented as *n* (%) or mean ± SD.

### 3.2 Clinical Efficacy

As shown in Table 3, statistically significant differences were observed between the observation and control groups in terms of cure, good, effective and ineffective rates (*p* < 0.001). The treatment effect in the observation group was markedly superior to that in the control group.

**TABLE 3:** Efficacy analysis of two groups.

Group	Cure, *n* (%)	Good, *n* (%)	Valid, *n* (%)	Invalid, *n* (%)
Observation group (*n* = 20)	5 (25%)	15 (75%)	0	0
Control group (*n* = 20)	2 (10%)	7 (35%)	5 (25%)	6 (30%)
*p*	0.001			

### 3.3 Vancouver Scar Scale (VSS)

As shown in Table 4, there were no statistically significant differences between the two groups in terms of color, thickness, vascular distribution, softness and total scores on the VSS prior to treatment (*p* > 0.05). Following treatment, however, the observation group exhibited significant differences from the control group in terms of color, vascular distribution, softness and total scores (*p* < 0.05), though thickness scores did not differ significantly (*p* > 0.05). Further analysis revealed that all VSS and total scores in both groups demonstrated statistically significant changes before and after treatment.

**TABLE 4:** Comparison of VSS scale scores between the two groups before and after treatment.

Group	Color score		Thickness score		Vascular distribution score		Softness score		VSS total score	
	Pre	Post	Pre	Post	Pre	Post	Pre	Post	Pre	Post
Observation group	2.75 ± 0.44	1.35 ± 0.99**	2.90 ± 0.72	1.30 ± 0.98**	2.70 ± 0.73	1.05 ± 0.76**	3.90 ± 0.72	1.65 ± 1.39**	12.25 ± 1.59	5.35 ± 3.58**
Control group	2.65 ± 0.49	2.10 ± 0.79^&^	3.00 ± 0.73	1.70 ± 0.80^&&^	2.50 ± 0.95	1.95 ± 0.76	3.95 ± 0.83	2.50 ± 1.24^&&^	12.10 ± 1.80	8.25 ± 2.73^&&^
*p*	0.503	0.012	1.141	0.165	0.460	0.01	0.839	0.048	0.782	0.006

*Note:* The postoperative score is the score after the end of follow‐up (24 m), which is the same as that after treatment mentioned below. Data are presented as *n* (%) or mean ± SD.

** *p* < 0.01 vs. pre (observation group).

^&^ *p* < 0.05 vs. pre (control group).

^&&^ *p* < 0.01 vs. pre (control group).

**FIGURE 2:**
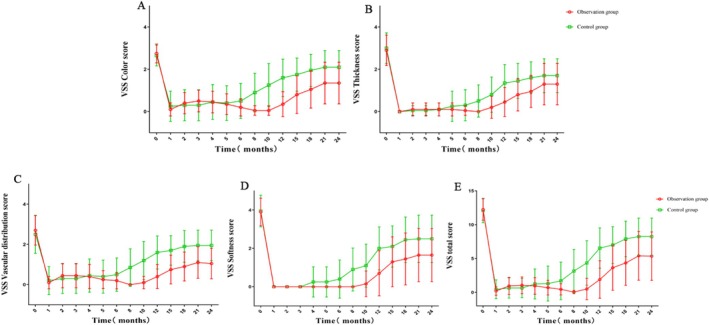
Comparison of VSS scores and analysis of time effects. (A) Color score; (B) Thickness score; (C) Vascular distribution score; (D) Softness score; (E) Total VSS score.

As shown in Figure 2, the control group reached minimum values for all VSS scores and total scores one month after treatment, subsequently increasing at varying rates. From the sixth month onwards, the month‐on‐month growth rates for all scores increased significantly, stabilizing around the 21st month when the month‐on‐month growth rates were almost zero. On average, total scores, color scores, thickness scores and softness scores reached approximately 50% of pretreatment values by the 12th month, while the average vascular distribution score peaked at around 50% of pretreatment values by the 10th month.

In the observation group, the total VSS score, thickness score, color score and vascular distribution score all increased gradually during the first month after treatment. This was followed by a decrease that reached its lowest point at eight months, after which there was a slight increase. The softness score remained at its lowest value until it began to rise from the tenth month onwards. From the 10th month onwards, the month‐on‐month growth rates for all scores increased significantly, ultimately stabilizing at around the 21st month with near‐zero month‐on‐month growth rates. Throughout the follow‐up period, the average total, color, thickness, softness, and vascular distribution scores in the observation group did not rise to 50% of their pretreatment values. Data analysis indicated an interaction effect between the treatment method and time. This suggests that, over time, improvements in the observation group were notably greater, as evidenced by smaller score increases compared to the control group.

### 3.4 Patient and Observer Scar Assessment Scale (POSAS)

As shown in Table 5, no statistically significant differences were observed between the two groups in terms of PSAS, OSAS, patient overall and observer total scores prior to treatment (*p* > 0.05). Following treatment, however, both groups exhibited statistically significant changes in PSAS, OSAS and patient overall scores compared to baseline (*p* < 0.05). Notably, the observation group had significantly lower PSAS and OSAS scores, as well as lower patient overall scores, after treatment than the control group (all *p* < 0.05), indicating the superior efficacy of the observation group.

**TABLE 5:** Comparison of POSAS scores between the two groups before and after treatment.

Group	PSAS score		OSAS score		Overall patient score		Overall observer score	
	Pre	Post	Pre	Post	Pre	Post	Pre	Post
Observation group	46.05 ± 4.696	16.70 ± 11.117**	47.50 ± 5.125	17.20 ± 11.162**	7.65 ± 0.813	2.90 ± 1.971**	8.05 ± 0.877	2.95 ± 1.877**
Control group	44.80 ± 5.681	26.95 ± 10.133^&&^	46.85 ± 5.851	28.35 ± 9.610^&&^	7.55 ± 1.050	4.50 ± 1.792^&&^	7.95 ± 1.050	4.85 ± 1.694^&&^
*p*	0.453	0.004	0.711	0.002	0.738	0.011	0.747	0.002

*Note:* The postoperative score is the score after the end of follow‐up (24 m), which is the same as that after treatment mentioned below. Data are presented as *n* (%) or mean ± SD.

** *p* < 0.01 vs. pre (observation group).

^&&^ *p* < 0.01 vs. pre (control group).

**FIGURE 3:**
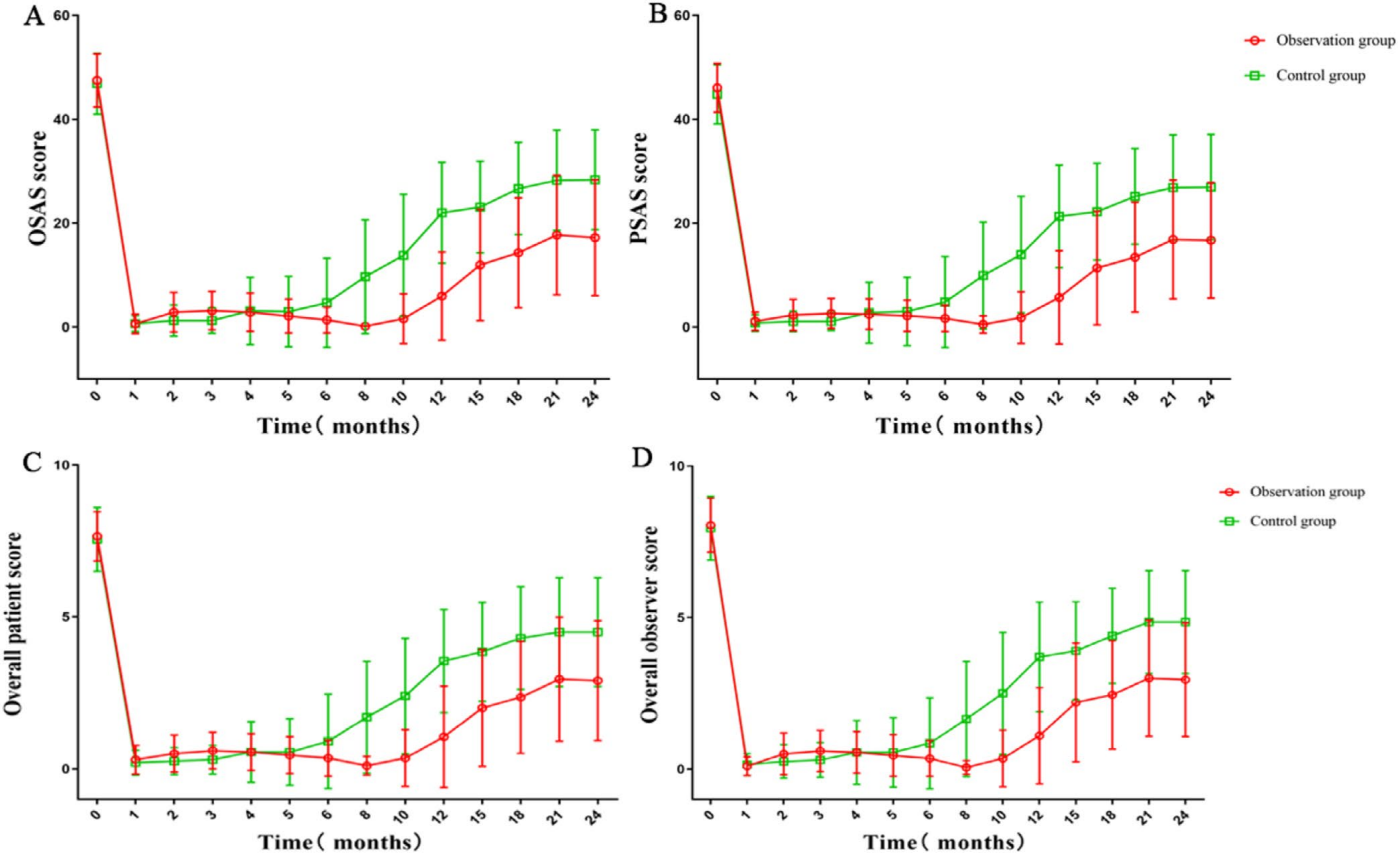
Comparison of POSAS scores and analysis of time effects. (A) POSAS score; (B) OSAS score; (C) Overall patient score; (D) Overall observer score.

As shown in Figure 3, the POSAS scores for the observation group were consistently lower than those for the control group, with this difference being statistically significant (*p* < 0.05). Assessments at different time points showed that the POSAS and total scores in the control group reached their minimum values one month after treatment. After this point, scores began to increase at varying rates. From the sixth month onwards, the month‐on‐month growth rates for all scores increased significantly, ultimately stabilizing around the 21st month when the month‐on‐month growth rate was almost zero. The average scores across all categories had risen to approximately 50% of their pretreatment values by the 12th month. In contrast, POSAS scores in the observation group increased gradually in the first month after treatment, followed by a decrease reaching its lowest level at eight months. Thereafter, there was a slight rise. From the tenth month onwards, significant increases in month‐on‐month growth rates were observed for all scores, stabilizing around the twenty‐first month with month‐on‐month growth rates approaching zero. Throughout the follow‐up period, the average scores for all categories in the observation group did not rise to 50% of the pretreatment values.

**FIGURE 4:**
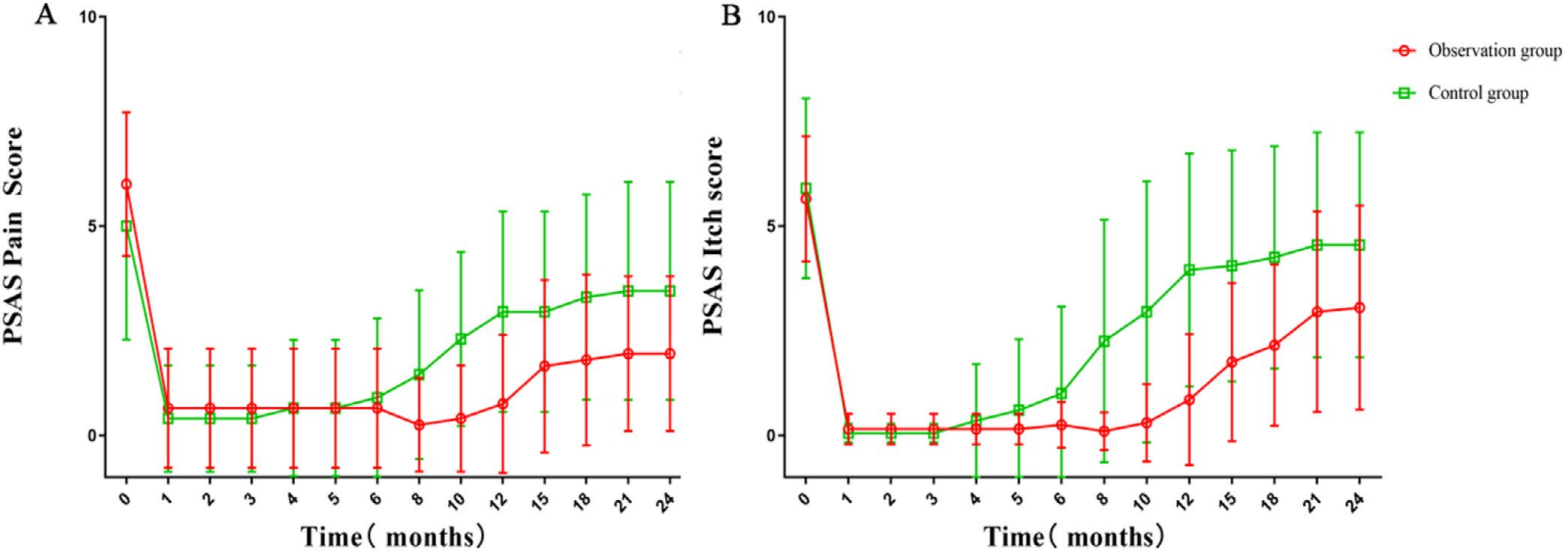
Comparison of PSAS pain and itch scores and analysis of time effects. (A) PSAS pain score; (B) PSAS itch score.

Figure 4 shows that there were significant differences in PSAS pain and itching scores between the two groups. The observation group reported consistently lower scores for both pain and itching posttreatment. Additionally, during the follow‐up period, the observation group maintained consistently lower score levels with minimal fluctuation. The data suggest an interaction effect between the treatment method and time: the improvements observed in the observation group increased significantly more over time than those seen in the control group.

### 3.5 Representative Cases of Changes Before and After Intervention in Both Groups

**FIGURE 5:**
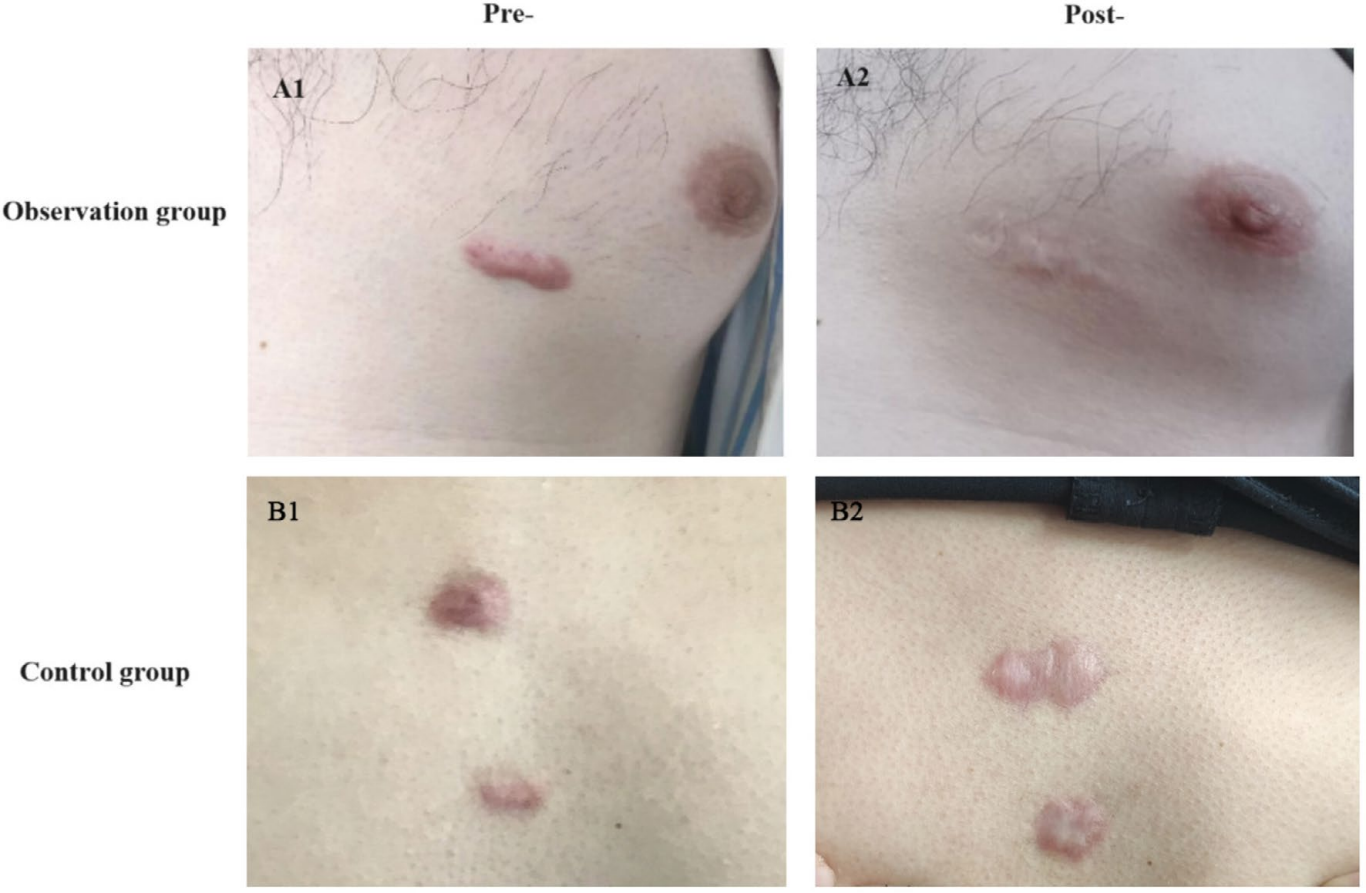
Visual changes in the skin before and after the intervention. (A1, A2) Observation group before and after treatment; (B1, B2) Control group before and after treatment.

As shown in Figure 5, one of the patients in the observation group was a 24‐year‐old male with a chest keloid that had been present for six years and had been accompanied by intermittent pain and itching for the previous year. The treatment regimen included tension‐releasing surgery, followed by postoperative radiotherapy and IPL treatment. Prior to treatment, the keloid appeared dark red with a hard texture, measuring 3 × 1 × 0.8 cm. The scar's surface was slightly rough, and it was significantly tender (A1). After 24 months of treatment, there was significant improvement in the appearance of the keloid, which became smoother and closer in color to normal skin tone. The texture was softened and there was no tenderness (A2).

In the control group, a 30‐year‐old male patient presented with a chest keloid that had been present for two years and was accompanied by intermittent pain and itching. The treatment regimen comprised tension‐releasing excision, followed by radiotherapy. A pretreatment examination revealed two keloids on the chest wall measuring 1.2 × 0.8 × 0.5 cm and 0.8 × 0.3 × 0.3 cm, respectively. Both keloids were dark red in color, firm in consistency, slightly rough in texture, had well‐defined borders and were tender to touch (B1). Twenty‐four months later, the keloids had faded to a pale pink color, softened in texture, and showed no signs of inflammation or active growth. The scars were stable, with smooth surfaces and no tenderness (B2).

In contrast to the control group patient, who only received surgical excision and radiotherapy, the patient in the observation group demonstrated significant improvement in the appearance of the keloid after undergoing tension‐releasing surgery combined with IPL treatment. The observation group experienced significantly greater improvements in appearance, texture and pain sensation, while changes in the keloids of the control group were minimal. These results suggest that combining IPL with radiotherapy is a more effective treatment for chest keloids in young and middle‐aged men.

## 4 Discussion

This study aimed to evaluate the effectiveness of combining postoperative radiotherapy with IPL for treating chest keloids in young and middle‐aged men. Quantitative assessment was carried out using the VSS and the Patient and POSAS. The results showed that, compared to the control group, the observation group had significantly better outcomes in terms of scar appearance and relief of associated symptoms, providing important evidence for the clinical application of IPL.

Keloids form primarily due to excessive production of the extracellular matrix, leading to disordered fibrotic collagen responses that extend beyond the original wound margins [11]. Despite the availability of treatment options, including medications and surgical interventions, keloids have a high recurrence rate [12]. Surgical excision combined with radiotherapy is widely recognized as an effective method of treating keloids, particularly in preventing recurrence [13]. However, this approach has limitations, including potential side effects associated with radiotherapy, which can induce psychological fear, particularly among younger patients. Some studies suggest that radiotherapy resistance may be related to pro‐fibrotic signals maintained by the surviving microvascular system [14]. In recent years, significant advancements in phototherapy technologies have introduced alternative approaches to keloid management via photophysical and photobiological mechanisms [15]. While promising, these methods often lack robust prospective controlled studies to support them, and direct comparative studies with surgical excision combined with radiotherapy remain scarce.

Compared to other phototherapy methods such as intense pulsed light (IPL) and lasers, IPL demonstrates greater targeting specificity [10]. With a wavelength designed within the range of 500–600 nm, it selectively acts on hemoglobin within blood vessels. This narrow spectrum minimizes melanin absorption in pigmented skin, thereby reducing the risk of hyperpigmentation compared to broad‐spectrum IPL (400–1200 nm) [16]. This mechanism induces thermal damage to endothelial cells, leading to vascular occlusion, reduced nutrient supply, and inhibition of keloid regeneration [17]. Furthermore, IPL promotes collagen remodeling by regulating the balance of MMP‐1/TIMP‐1 [18] and modulating cellular proliferation and apoptosis [19], thereby addressing abnormalities in collagen metabolism. In this prospective clinical trial, significant improvements in various scores were observed in the treatment group posttreatment, particularly with regard to scar pigmentation, vascular distribution and pliability. These results imply that IPL has great potential in modulating the deep vascular and surrounding tissue components of scars.

Interestingly, this study observed an ongoing improvement in scores for the intervention group, particularly a significant reduction in pain and itchiness. This symptom relief may be linked to IPL's suppression of neuropeptide release from keloid‐associated sensory nerves [20]. This suggests that when combined with radiotherapy, IPL effectively alleviates keloid symptoms and enhances patients' quality of life. During the follow‐up period, the observation group showed minimal fluctuations in VSS and POSAS scores, with substantial improvement occurring after 10 months. This suggests that IPL could be pivotal in the long‐term management of keloids, reducing the risk of recurrence. We believe that combining IPL with postoperative radiotherapy enhances its efficacy. Surgical excision quickly removes the bulk of the keloid; radiotherapy effectively inhibits excessive fibroblast proliferation; and IPL, through its unique photothermal effects, improves scar pigmentation and pliability, reduces abnormal vascular proliferation and alleviates symptoms such as itching and pain. The addition of IPL may also reduce the required radiotherapy dosage, thereby lowering the risk of radiotherapy‐related side effects.

Although this study demonstrated the significant efficacy of IPL, certain limitations remain. The relatively small sample size and restriction of the study population to males aged 20–30 years limit the generalizability of the findings. Future studies should be expanded to include patients of different ages, sexes and keloid locations in order to validate these results. Furthermore, although the observation group exhibited a low risk of recurrence during the 21‐month follow‐up period, the underlying biological mechanisms warrant further investigation.

## 5 Conclusion

In conclusion, a combination of postoperative radiotherapy and IPL has been shown to be highly effective in treating chest keloids in young and middle‐aged male patients. This combination improves the appearance of scars and effectively alleviates symptoms such as pain and itching, demonstrating substantial clinical utility. IPL's targeted and photothermal mechanisms complement the shortcomings of standalone radiotherapy. Future research should focus on optimizing treatment protocols and exploring the efficacy and mechanisms of this approach for different scar types to further improve clinical outcomes.

## Author Contributions

Shengping Chen; experiments: Qiaoling Weng, Junjun Sang; data analysis/writing: Qiaoling Weng, Jie Wu, Wen Xu; manuscript revision: Xiangqi Chen, Junjun Sang; All authors approved this final version.

## Ethics Statement

We conducted a prospective randomized controlled trial, selecting a total of 52 male patients with chest keloids who visited The 900th Hospital of Joint Logistic Support Force between March 2018 and March 2019. The study was approved by the Joint Logistics Support Force 900th Hospital Ethics Committee (2017‐030) and performed by the principles of the revised Declaration of Helsinki.

## Conflicts of Interest

The authors declare no conflicts of interest.

## Supporting information


**Data S1:** jocd70567‐sup‐0001‐DataS1.xlsx.

## Data Availability

The data that supports the findings of this study is available in the Supporting Information of this article.
